# Global genomic diversity and conservation priorities for domestic animals are associated with the economies of their regions of origin

**DOI:** 10.1038/s41598-018-30061-0

**Published:** 2018-08-03

**Authors:** Min Zhang, Wei-Feng Peng, Xiao-Ju Hu, Yong-Xin Zhao, Feng-Hua Lv, Ji Yang

**Affiliations:** 1 School of Life Sciences, University of Science and Technology of China, Hefei, Anhui 230027 China; 2 CAS Key Laboratory of Animal Ecology and Conservation Biology, Institute of Zoology, Chinese Academy of Sciences (CAS), Beijing, 100101 China; 3 University of Chinese Academy of Sciences (UCAS), Beijing, 100049 China

## Abstract

Domestic animals play a key role in human survival and the development of civilization. However, the genetic resources of domestic animals are facing an alarming rate of erosion due to socioeconomic changes, economic globalization and financial constraints. In this study, through genome-wide SNP analysis, we estimated the heterozygosity, inbreeding coefficient, effective population size, and runs of homozygosity to identify the breeds facing the risk of extinction for sheep and cattle across the world. In particular, we quantified the contribution of 97 sheep breeds and 53 cattle breeds to genomic diversity (within-breed, between-breed and total) and prioritized the breeds for conservation. Additionally, we compared the average values of genomic diversity between breeds from regions (or countries) in different economic categories (underdeveloped, developing and developed), and found that breeds in developed regions exhibit significantly higher levels of total genomic diversity than those in underdeveloped and developing regions. Altogether, our results suggested that conservation priority should be given to breeds in developed regions to secure the future genomic diversity hotspots of domestic animal resources.

## Introduction

Farm animals contribute in many ways to human survival and economic development, particularly as societies evolve from subsistence agriculture into market-based economies^1^. However, in the past two decades, farm animal genetic resources have faced an alarming rate of extinction and genetic erosion, as approximately 200 uniquely adapted breeds have become extinct and up to 30% of global livestock breeds (approximately 1,200–1,500) are currently endangered^2^. This loss of diversity is attributed to multiple causes, including regional economic forces such as the decline in the economic viability of traditional livestock production systems^3^, destruction of the native habitats (e.g., grazing lands) of livestock breeds^4^, rapid dissemination of uniform and highly productive breeds at the expense of native genetic resources^5^ and the extension of markets and economic globalization^6^. In this context, an integrated analysis of the genomic diversity of farm animals and the associated economies can aid an understanding of economic impact on the changes in farm animal genomic diversity in recent years. Additionally, it will contribute to the development of governmental interventions and the implementation of conservation priorities that are necessary to ensure that ongoing agricultural development will be compatible with the conservation and sustainable use of local livestock diversity^7^.

Here, we ranked the conservation priorities of 97 native sheep breeds and 53 native cattle breeds from around the world based on genome-wide single-nucleotide polymorphism (SNP) data for the first time. Firstly, we calculated heterozygosity, inbreeding coefficient, and runs of homozygosity (ROH) for each sheep and cattle breed to detect the small and inbred populations that are facing the risk of extinction. Moreover, we implemented the first association analysis between economic indexes and genomic diversity by studying a total of 2,941 animals from 150 native breeds of two globally distributed livestock species: sheep (1,910 animals, 97 breeds; Supplementary Table 1) and cattle (1,031 animals, 53 breeds; Supplementary Table 2). Specifically, the estimations of genomic diversity contributions for sheep and cattle were based on SNP array data genotyped from the Ovine SNP50 (or Infinium HD) and Bovine SNP50 BeadChips, respectively. Through the molecular coancestry^8^ and allelic richness approaches^9^, we calculated the breed contribution to genomic diversity at three levels: within-population diversity (ΔGD_WS_), between-population diversity (ΔGD_BS_), and total genomic diversity (ΔGD_T_) for each breed after its removal (Supplementary Tables 1 and 2). For region-wide breeds, the estimation of genomic diversity contributions was implemented by excluding all of the breeds within the region from the total breeds (Supplementary Tables 3 and 4). We then compared the ΔGD_WS_, ΔGD_BS_ and ΔGD_T_ among the regions of origin (or countries) in terms of the annual gross domestic product (GDP) per capita (GDPPC). We also took into account the increase in the GDP per capita (ΔGDPPC) for the regions during the past two decades in a dual-regression analysis to correlate the contribution of the genomic diversity of the breeds and the past economic development in a region. To facilitate comparisons among the regions with different economies, we grouped the regions into three categories (i.e., underdeveloped, developing and developed) according to the average GDPPC (A-GDPPC) during the years 1993–2013. Based on all the above analyses, we were finally able to determine the contribution of each breed to the maximum amount of genomic diversity and establish the core sets of breeds with higher priority for conservation.

## Results

### Genetic diversity pattern and inbreeding coefficient

Heterozygosity is a measure of genetic variation within a population. We estimated the observed (*H*_O_) and expected (*H*_E_) heterozygosity for each sheep and cattle breed. For sheep breeds, the highest *H*_O_ value was observed in the RAA (0.394) breed from Spain, and the lowest was in the SPS breed (0.252) from Yunnan province of China. The highest and lowest expected (*H*_E_) heterozygosity were also observed in the RAA breed (0.385) and SPS breeds (0.258), respectively (Supplementary Table 1). For the cattle breeds, CANC breed from Brazil and PRP breed from France had the highest *H*_O_ (0.362) and *H*_E_ (0.343) values, respectively, and BALI breed from Indonesia presented the lowest *H*_O_ (0.061) and *H*_E_ (0.063) values (Supplementary Table 2). For the estimation of inbreeding coefficients (*F*_IS_) of sheep breeds, BGE breed had the highest *F*_IS_ value (0.157) and DSH breed had the lowest *F*_IS_ value (−0.065) (Supplementary Table 1). In cattle breeds, the SOM (0.050) and ND2 (−0.108) presented the highest and lowest *F*_IS_ values, respectively (Supplementary Table 2).

### Effective population size

**Figure 1:**
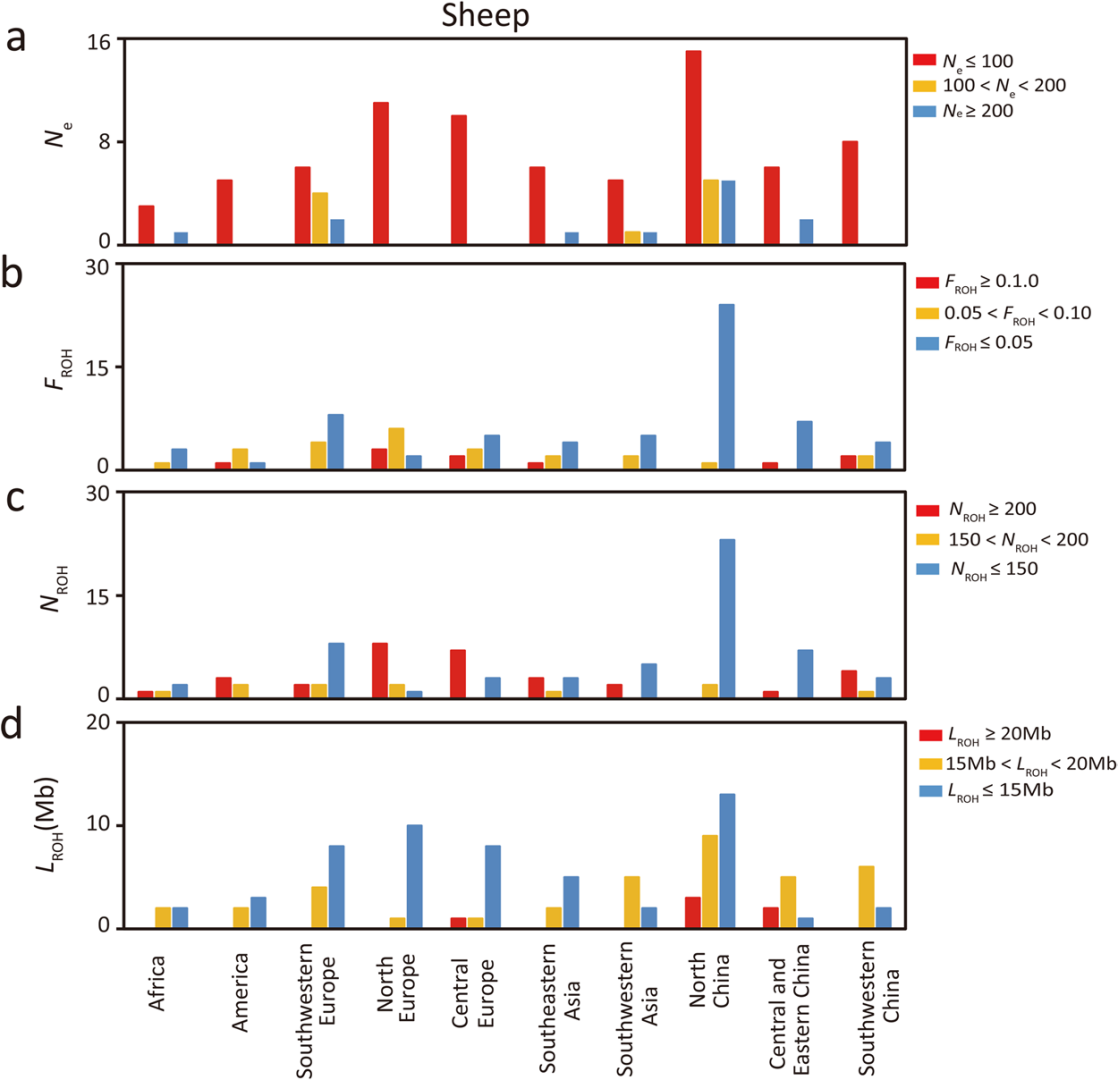
Distribution patterns of *N*_e_ (**a**) N_ROH_ (**b**) L_ROH_ (**c**) F_ROH_ (**d**) found in 97 sheep breeds. *N*_e_: effective population size. F_ROH_: inbreeding coefficient based on runs of homozygosity. N_ROH_: number of ROH. L_ROH_: mean length of ROH. The Y axis represents the number of breeds that fall into three different ranges of the statistics.

**Figure 2:**
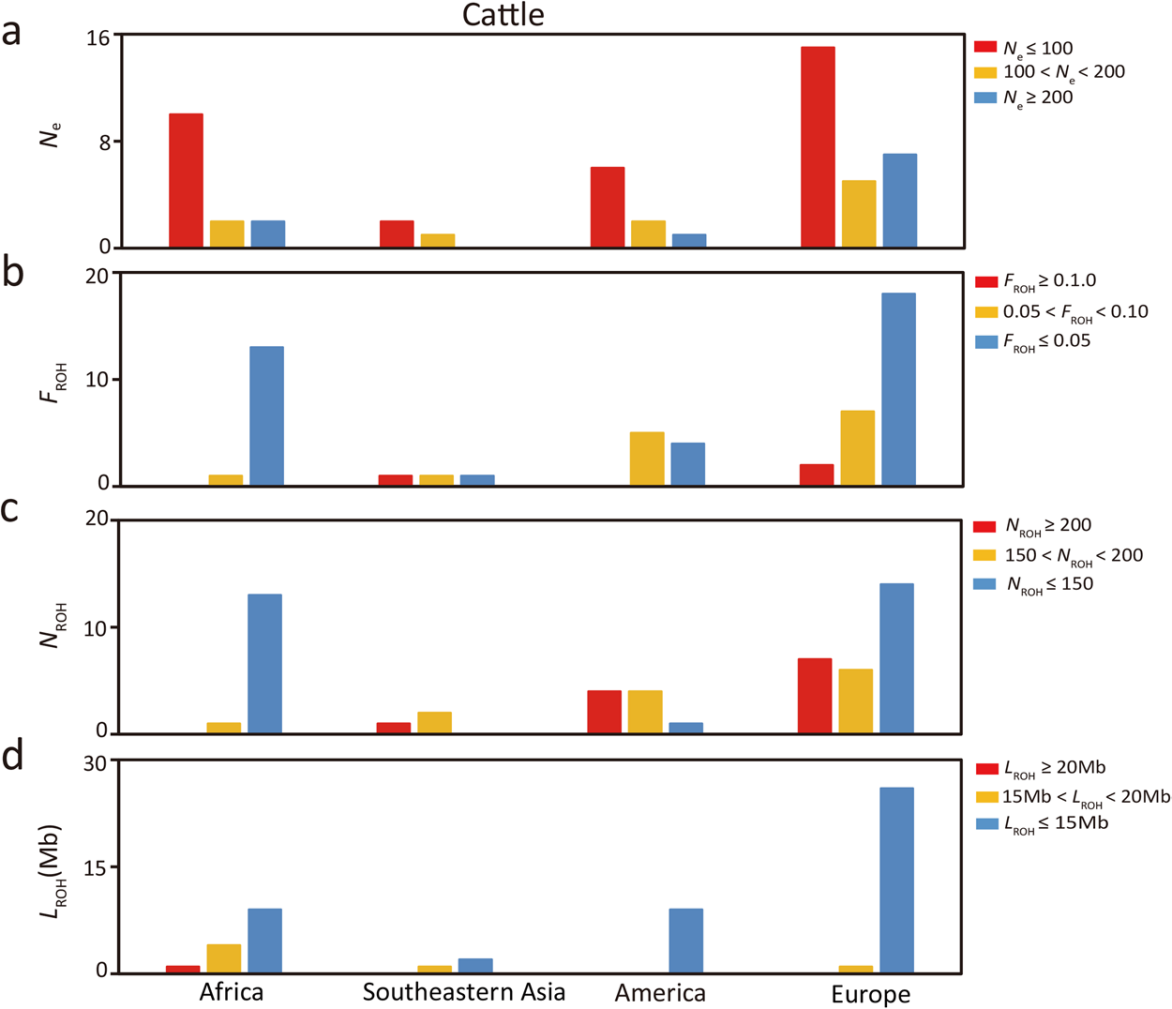
Distribution patterns of *N*_e_ (**a**) N_ROH_ (**b**) L_ROH_ (**c**) F_ROH_ (**d**) found in 53 cattle breeds. *N*_e_: effective population size. F_ROH_: inbreeding coefficient based on runs of homozygosity. N_ROH_: number of ROH. L_ROH_: mean length of ROH. The Y axis represents the number of breeds that fall into three different ranges of the statistics.

The results for effective population size (*N*_e_) for sheep and cattle were calculated based on the linkage disequilibrium method and presented in the Supplementary Tables 1 and 2. In sheep, *N*_e_ ranged from 3,074 (BYK) down to 3 (WNS), while in cattle the value ranged from 1,429 (ZBO) down to 1 (BALI). The high degree of inbreeding may be the reason for the extremely low *N*_e_ value of BALI, as evidenced by the high *F*_IS_ and *F*_ROH_ value observed in this breed (Supplementary Table 2). To glean further details, we divided the *N*_e_ values into three categories: *N*_e_ ≤ 100, 100 < *N*_e_ < 200, and *N*_e_ ≥ 200, and the number of breeds within each of the three categories were summarized in Table 1 (also see Figs 1a and 2a, Supplementary Figs 1a and 2a and Supplementary Tables 1 and 2).

**Table 1:** The number of sheep and cattle breeds in different categories of population genetic parameters.

Parameters	Categories	Number of breeds	
		Sheep	Cattle
*N*_e_	*N*_e_ ≤ 100	75	33
	100 < *N*_e_ < 200	10	10
	*N*_e_ ≥ 200	12	10
F_ROH_	F_ROH_ ≥ 0.1	10	3
	0.05 < F_ROH_ < 0.1	24	14
	F_ROH_ ≤ 0.05	63	36
N_ROH_	N_ROH_ ≥ 200	31	12
	150 < N_ROH_ < 200	11	13
	N_ROH_ ≤ 150	55	28
L_ROH_	L_ROH_ ≥ 20	6	1
	15 < L_ROH_ < 20	37	6
	L_ROH_ ≤ 15	54	46

### Runs of homozygosity (ROH)

In this study, we calculated the ROH statistics for each breed of sheep and cattle, and subsequently the inbreeding coefficient based on ROH (F_ROH_) was estimated. The F_ROH_ values ranged from 0.0004 (WZS) to 0.2016 (WIL) in sheep breeds (Supplementary Table 1) and from 0.0001 (ZEB) to 0.6842 (BALI) in cattle breeds (Supplementary Table 2). Regarding the number of ROH (N_ROH_) above 500 kb, the sheep breed of WIL (N_ROH_ = 780) showed the highest N_ROH_ value and WZS (N_ROH_ = 2) had the lowest N_ROH_ value (Supplementary Table 1). For the cattle breeds, ZEB (N_ROH_ = 1) and BALI (N_ROH_ = 1,127) breeds showed the lowest and highest N_ROH_ value, respectively (Supplementary Table 2). Finally, we also calculated the average length of ROH above 500 kb for each breed (L_ROH_). For sheep and cattle breeds, L_ROH_ values ranged from 26.9 Mb (HLS) to 9.0 Mb (SOA), and 21.8 Mb (BOR) to 5.1 Mb (ZEB), respectively (Supplementary Tables 1 and 2). To obtain more details, F_ROH_, N_ROH_ and L_ROH_ values were also divided into three categories: F_ROH_ categories (F_ROH_ ≥ 0.10, 0.05 < F_ROH_ < 0.10, and F_ROH_ ≤ 0.05), N_ROH_ categories (N_ROH_ ≥ 200, 150 < N_ROH_ < 200, and N_ROH_ ≤ 150) and L_ROH_ categories (L_ROH_ ≥ 20 Mb, 15 Mb < L_ROH_ < 20 Mb, and L_ROH_ ≤ 15 Mb), and the number of breeds belong to each of the categories were shown in Table 1 (also see Figs 1 and 2, Supplementary Figs 1 and 2 and Supplementary Tables 1 and 2).

### Genetic structure

The relationship between breeds was investigated using the NJ tree based on the Reynolds distance estimated from allele frequency differences, and branch lengths were highly variable. The 97 sheep breeds were clearly divided into African, Asian (Southwestern Asian, Southeastern Asian, and China), and European sheep breeds (Supplementary Fig. 3a). However, sheep from the Americas were clustered with European-derived sheep breeds (Supplementary Fig. 3a). The 53 cattle breeds were clearly divided into European and African cattle breeds, although the American and Southeastern Asian cattle breeds exhibited an admixture with cattle breeds from Europe and Africa (Supplementary Fig. 3b).

### Conservation priorities

The mean values obtained using the molecular coancestry and allelic richness methods showed ranges for ΔGD_WS_, ΔGD_BS_, and ΔGD_T_ in sheep from −0.200 (SPS) to 0.077 (RAA), −0.067 (KAZ) to 0.218 (SOA), and −0.044 (SPS) to 0.042 (DSH), respectively (Supplementary Table 1). In cattle, these values ranged from −0.781 (BALI) to 0.257 (CANC), −0.198 (CANC) to 0.670 (BALI) and −0.176 (LAG) to 0.172 (HFD), respectively (Supplementary Table 2).

As representative of the main cattle breeds reared around the world, six cattle breeds (HFD, HO, AN, ANR, NRC, and BEFM; 11.3%) showed a ΔGD_T_ > 0.1 and 11 (20.8%) breeds showed a ΔGD_T_ < −0.1 in this study (Supplementary Table 2 and Supplementary Fig. 4f). However, none of the native sheep breeds presented extreme values of ΔGD_T_ > 0.1 or <−0.1 (Supplementary Table 1 and Supplementary Fig. 4c). This observation implied a more diverse genetic constitution of sheep compared with cattle. Another possible reason is the differences in number of sheep and cattle breeds analyzed, as the contribution of a single breed to genomic diversity is expected to be lower in a larger sample.

### Comparison of conservation priorities in three different economic categories

**Figure 3:**
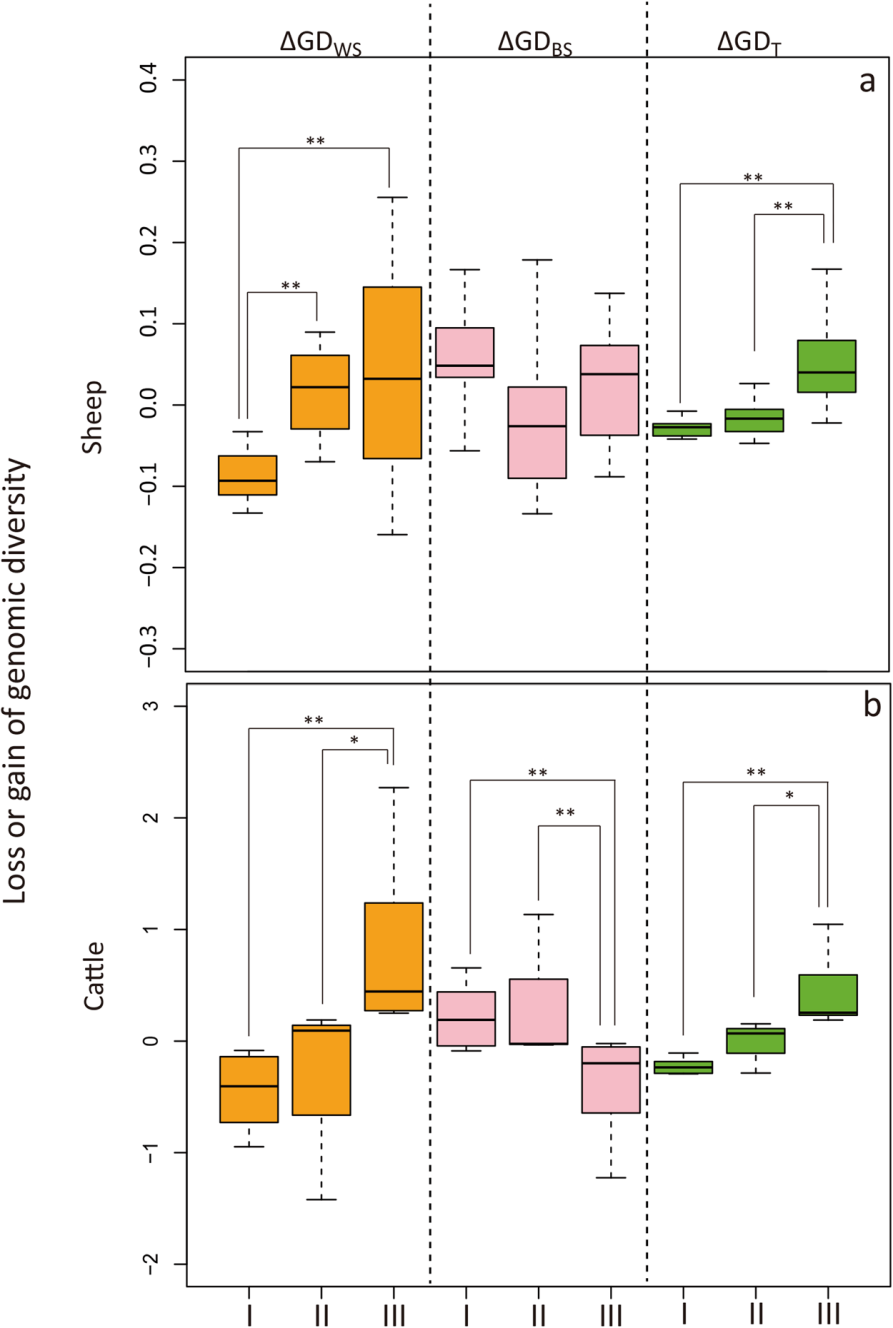
Comparisons of the genomic diversity contributions of sheep (**a**) and cattle (**b**) breeds between regions (or countries) within the three economic categories. Three economic categories: I, underdeveloped; II, developing; III, developed. Three genomic diversity contributions: within-population, ΔGD_WS_ (yellow); between-population, ΔGD_BS_ (pink); total genomic diversity, ΔGD_T_ (green). **P < 0.01; *P < 0.05. The loss or gain of genomic diversity is indicated by the average values of estimates obtained using the molecular coancestry and allelic richness methods.

**Figure 4:**
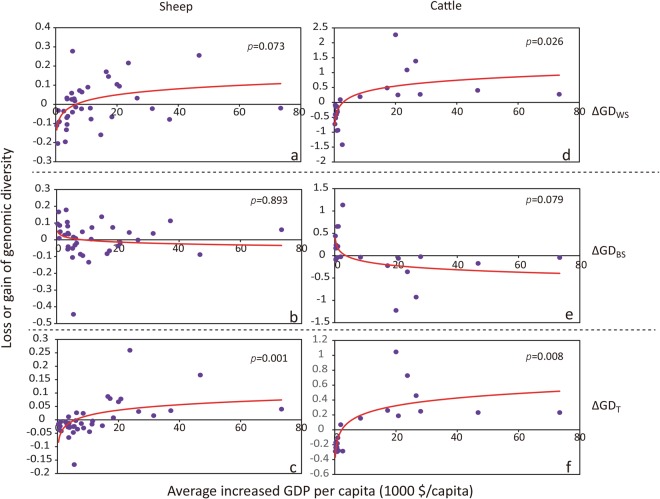
Regression between the genomic diversity contributions and the increase in GDP per capita (ΔGDPPC) during the years 1993–2013. (**a**–**c**) Within-population (ΔGD_WS_), *R*^2^ = 0.087, *P* > 0.05; between-population (ΔGD_BS_), *R*^2^ = 0.001, *P* > 0.05; total genomic diversity (ΔGD_T_), *R*^2^ = 0.278, *P* < 0.01 in sheep. (**d**–**f**) Within-population (ΔGD_WS_), *R*^2^ = 0.0235, *P* < 0.05; between subpopulation (ΔGD_BS_), *R*^2^ = 0.153, *P* > 0.05; total genetic diversity (ΔGD_T_), *R*^2^ = 0.318, *P* < 0.01 in cattle. The loss or gain of genomic diversity is indicated by the average values of the estimates obtained using the molecular coancestry and allelic richness methods.

According to the average GDP per capita (A-GDPPC) during 1993 to 2013, we divided countries or regions into three economic categories: underdeveloped countries/regions, developing countries/regions, and developed countries/regions. For sheep, English breeds showed the highest ΔGD_T_ (0.259) value, Xinjiang breeds showed the highest ΔGD_WS_ (0.278) value, and South African breeds were observed with the highest ΔGD_BS_ (0.179) value. In contrast, Bangladeshi breeds showed the lowest ΔGD_WS_ (−0.205) value, and Xinjiang breeds had the lowest ΔGD_BS_ (−0.445) and ΔGD_T_ (−0.167) values (Supplementary Table 3). For cattle, ΔGD_WS_ ranged from 2.271 (France) to −1.421 (Indonesia), ΔGD_BS_ ranged 1.134 (Indonesia) to −1.225 (France), and ΔGD_T_ ranged from 1.047 (France) to −0.293 (Pakistan) (Supplementary Table 4). Further comparisons revealed significant differences in ΔGD_WS_ and ΔGD_T_ between breeds from the three economic categories. For instance, both species showed that the breeds from developed regions had the highest average value of ΔGD_T_, followed by those from developing regions and underdeveloped regions (Fig. 3). In addition, the breeds in developing and developed regions exhibited significantly (*P* < 0.05) higher ΔGD_WS_ values than those in underdeveloped regions, whereas the ΔGD_BS_ estimates for breeds from different regions within the three economic categories were not significantly different from each other (Fig. 3a). We also examined the impact of economic growth, which is typically measured as the rate of change in the average annual GDP per capita (ΔGDPPC), on the gain or loss of genomic diversity. Our results showed that ΔGD_WS_ and ΔGD_T_ presented significant associations with ΔGDPPC during the past two decades (sheep: *R*^2^ = 0.087, *P* = 0.073 (ΔGD_WS_), *R*^2^ = 0.278, *P* < 0.01 (ΔGD_T_); cattle: *R*^2^ = 0.235, *P* < 0.05 (ΔGD_WS_); *R*^2^ = 0.318, *P* < 0.01 (ΔGD_T_); Fig. 4a,c,d,f). In both species, ΔGD_WS_ and ΔGD_T_ displayed a rapid increase for ΔGDPPC < 20,000 and a slow increase thereafter (Fig. 4).

## Discussion

Heterozygosity is an important factor for estimating the genetic variability in domestic animals. Heterozygosity represents the genetic potential and adaptation abilities to the natural environment^10^. In this study, we found that the livestock in developing and developed regions have higher heterozygosity than those in underdeveloped regions or some island populations. For example, sheep breeds from Southwestern Europe and Northern China obviously harbor higher observed (*H*_O_) and expected (*H*_E_) heterozygosity than those from Africa and southwestern China, which belong to underdeveloped regions (Supplementary Table 1). Similarly, cattle breeds from Europe and America possess higher *H*_O_ and *H*_E_ values than those from Africa and Asia (Supplementary Table 2). Besides economic factors, another reasonable explanation is that breeds with high heterozygosity are from regions near the domestication center or trade routes^11–15^. Notably, we found that the island breeds always show low heterozygosity. For example, BALI breed, which is distributed on the Bali island of Indonesia, exhibits the lowest heterozygosity (*H*_O_ = 0.061 and *H*_E_ = 0.063). Also, GNS (*H*_O_ = 0.299 and *H*_E_ = 0.297) and JER (*H*_O_ = 0.290 and *H*_E_ = 0.280) breeds from Guernsey and Jersey islands near England harbor lower heterozygosity than breeds from the European continent (Supplementary Table 2). These results suggested that these island breeds have suffered population decline and their genetic variation has been reduced.

Effective population size (*N*_e_) has been regarded as a popular parameter to investigate small populations and endangered species. According to the 50/500 rule of thumb^16^, *N*_e_ values under 50 means that the population is likely to face a serious genetic threat after 5 or more generations. This is because small populations cannot easily avoid inbreeding depression. *N*_e_ values above 500 means that the population can maintain an evolutionary potential for longer periods of time. However, Frankham *et al*.^17^ have proposed a new criterion that *N*_e_ values under 100 and above 1,000 are suitable to control inbreeding and maintain high genetic diversity, respectively^17^. Based on the new criterion, more than half of the breeds for both species in this study are facing the risk of extinction as their *N*_e_ values were under 100 (Figs 1a and 2a, Supplementary Figs 1a and 2a and Supplementary Tables 1 and 2). The probable cause of this observation was human intervention in the process of livestock development, such as introduction of breeds and intensive breeding^18–22^. Overall, we can see that most sheep and cattle breeds do not show a long-term stable status, and thus, we need to pay more attention and carry out appropriate conservation strategies on these native breeds with small effective population size.

ROH is a key parameter to detect the level of matings between related individuals in a closed or small population, and the number and length of ROH could indicate the extent of recent inbreeding and artificial selection^23,24^. Because of its utility, ROH has been used to investigate genome-wide autozygosity regions associated with some important traits and diseases in domestic animals and humans^25,26^. Inbreeding is among the major causes that lead to an increase in homozygosity, which in turn, reduce fitness and accumulate more recessive alleles that may lead to expression of unfavorable phenotypes^27,28^. According to the inbreeding coefficient based on runs of homozygosity (F_ROH_), we have identified 10 sheep breeds (WIL, BOR, WNS, BCS, ZTS, BGE, DSH, BHM, EFB and YXZ) and three cattle breeds (BALI, SH and JER) with an F_ROH_ value above 0.10. In addition, these breeds also showed a large number of runs of homozygosity (N_ROH_ ≥ 200) (Supplementary Tables 1 and 2). Together these results suggest that these breeds were founded as small populations or the populations recently experienced severe inbreeding. Previous studies have provided evidence that domestic animals have suffered serious inbreeding and the effective population size is declining under the pressure of artificial selection^29,30^. In our study, most of the sheep and cattle breeds had a ROH length above 10 Mb, suggesting they share recent ancestors 3 generations ago^31,32^. Also, there was a negative correlation between F_ROH_ and *N*_e_, especially in the sheep breeds. Sheep breeds from North China had a lower F_ROH_ and a higher *N*_e_ than other regions, while in cattle breeds the lowest F_ROH_ and the highest *N*_e_ appeared in Africa and Europe. Interestingly, although some sheep and cattle breeds from the developed regions show high F_ROH,_ N_ROH_ and L_ROH_ and low *N*_e_ values, they make contributions to genetic diversity. For example, 6 sheep breeds (BOR, DSH, ISF, SOA, WIL and GAL) have a *N*_e_ value under 200, but they exhibit high ΔGD_BS_ and positive ΔGD_T_ values (Supplementary Table 1). Also, three cattle breeds (SH, GNS and JER) present the same pattern as the sheep breeds. Therefore, these breeds should be subjected to conservation measures to avoid the erosion of their genetic diversity. In addition, the breeds from developing and underdeveloped regions with low *N*_e_ values, such as sheep breeds BGE, GAR, ZTS and SPS, and cattle breeds LAG, ND2, BOR, ZMA and BALI (Supplementary Tables 1 and 2), are strongly differentiated from the continental breeds (Supplementary Fig. 3), implying that they may have some special economic traits to be used in breeding programs.

Population structure is a useful information for humans to design effective strategies to improve the conservation of farm animal genetic resources. In this study, we performed a comprehensive analysis of population structure of sheep and cattle breeds worldwide. The 97 sheep breeds can be clearly divided into six geographic regions (Europe, Southwestern Asia, Southeastern Asia, China, America and Africa) (Supplementary Fig. 3a). Previous research on 74 diverse sheep breeds collected globally documented a high level of admixture within the European sheep, which showed high co-ancestry with most other breeds^33^. Compared to the previous study, our data set incorporated sheep breeds from China, and identified the breeds with large distinctness among the sheep breeds. Based on the results of the NJ tree, the breeds from Southeastern Asia, Africa, Southwestern China and several breeds (WIL, BRL, DSH, EFB, ISF and GAL) from Europe have longer branches (Supplementary Fig. 3a), suggesting these breeds are isolated breeds and possess unique genetic characteristics. The investigation of genetic structure of cattle reveals that cattle have two domestication centers: Fertile Crescent and Indus valley^15^. The African taurine has large divergence from European taurine and Asian indicine because of their large portion of wild African auroch ancestry^34^. This is consistent with our results that African cattle show apparent divergence from European cattle (Supplementary Fig. 3b). In addition, 7 African (LAG, BAO, ND2, ZMA, NDAM, SOM and ZEB) and 3 Southeastern Asian breeds (SAHW, GIR and BALI) make a large contribution to the between-breed genetic diversity (ΔGD_BS_) (Supplementary Table 2). These results reflect the unique genetic resources carried by these breeds.

From the results of loss or gain of genetic diversity, coupled with the evidence of the above statistics (i.e., heterozygosity, *N*_e_, ROH and population structure), our most prominent finding was that breeds from developed regions exhibit higher total genomic diversity than those in underdeveloped and developing regions, and thus, should be given a higher conservation priority (Fig. 3). Since the sample of this study does not cover the global distributions of sheep and cattle, future studies based on more extensive and representative samples are needed to verify this finding. For the potential factors affecting conservation priority, whether the economic situation measured based on the annual GDP per capita plays a direct role in livestock conservation is unclear. One hypothesis is that a better economy induces more effective conservation measures, including higher subsidies for raising local livestock breeds and more financial investment in conservation programs. For example, the European Union has long recognized the importance of conserving animal genetic resources (AnGR) and, in 1992, initiated a policy of economic incentives for farmers keeping native breeds, under EC regulations 2078/92 and 1257/99^35^. However, breeds from underdeveloped regions, such as African countries (EMZ, NQA, RMA and RDA) and the Yunnan-Kweichow Plateau of China (ZTS, WGS, SPS, TCS, DQS, NLS and WNS), where little or no financial support has been provided for local livestock conservation measures, showed extreme negative values of within-breed genomic diversity (Supplementary Table 1 and Supplementary Fig. 4a). This observation could be due to factors such as long-term genetic isolation, genetic drift, or inbreeding, and thus, effective conservation measures preventing genetic drift in small-sized and isolated breeds and promoting reasonable gene flow with outside breeds should be implemented for the native breeds in underdeveloped regions. A second hypothesis is that economic growth is one causative factor for the change in the livestock production system, possibly through effects on breeding programs. Rapid economic growth could have led to an increased need for more productive and synthetic livestock breeds^36^ and therefore contributed to the increased ΔGD_T_ and ΔGD_WS_ and decreased ΔGD_BS_ values observed in livestock (Fig. 4). Native breeds in developed regions exhibited high values of both ΔGD_WS_ and ΔGD_T_ (Fig. 3), which could also be due to the improvement of breeding programs by avoiding indiscriminate crossbreeding^37^. However, breeds in developing regions such as Xinjiang of China showed high values of ΔGD_WS_ but negative values of ΔGD_T_ (Supplementary Table 1 and Supplementary Fig. 4a,c), which could be explained by the development scheme of extensive and intensive crossbreeding of sheep with exotic breeds adopted in past decades^38^. Apart from the potential impact of economic situation on livestock conservation, several other factors may also account for the conservation status of domestic animals. For instance, the development of the breed concept, which originated 200–250 years ago in Europe, has probably affected the number of breeds differentiated and selected within the region. Also, transboundary exchanges of breeds may have played a fundamental role in shaping the genetic diversity of breeds, especially in American and Australian countries.

Another important finding regarding genetic diversity was that breeds in developing regions have been facing the erosion of between-breed genomic diversity (ΔGD_BS_). Both species are transboundary animals, and many highly productive commercial breeds have been transferred worldwide to upgrade local breeds^39–42^. With the rapid economic development and increase in the human population in developing regions, the share of trade in genetic material from developed to developing countries increased from 20% in 1995 to 30% in 2005^43,44^. Long-term intensive crossbreeding programs using imported exotic breeds have narrowed the diversity of local genetic materials and led to the loss of between-breed genomic diversity in developing regions^45^. In northern China, the estimates of both ΔGD_BS_ and ΔGD_T_ were negative, suggesting that northern Chinese sheep have suffered severe genetic erosion induced by exotic breeds in the past, which has resulted in the loss of both total and within-breed genomic diversity (Supplementary Fig. 4b,c). Similar scenarios were also observed in the breeds of Iran, Turkey and Pakistan (Supplementary Fig. 4a,c). However, livestock in underdeveloped regions (including remote and isolated island areas) are commonly used to service the local community^46,47^, being subjected to little or no influence from exotic breeds, and these populations therefore showed high ΔGD_BS_ values (Supplementary Fig. 4b,e). For example, the diverse and isolated topography of the Yunnan-Kweichow Plateau of China has contributed to the genetically isolated populations of local sheep breeds, which show high and positive ΔGD_BS_ values, but negative ΔGD_T_ values (Supplementary Fig. 4b,c). Similar results were observed in cattle from mainland Africa and Madagascar (Supplementary Fig. 4e,f). In these scenarios, breeds with high levels of within-breed genomic diversity (ΔGD_WS_) should be ranked with high conservation priorities to preserve closed populations that will be capable of coping with future challenging environments or diversified food production. However, breeds with large divergence should also give protection measures to prevent their extinction due to their unique genetic resources and special adaptation to local environment.

As the ovine and bovine SNP BeadChips have generally been developed on European breeds, it is expected that ascertainment bias would impact the estimates of genetic diversity metrics. However, our results should not be significantly affected by ascertainment bias for the following reasons. On the one hand, Herráez *et al*.^48^ have demonstrated that the effect of ascertainment bias could be countered if the SNPs with high LD were removed. Based on this finding, we recalculated the heterozygosity and ΔGD_T_ (loss or gain of genetic diversity) by using the LD-pruned data, and obtained similar results with those not controlling for LD (Supplementary Fig. 5). On the other hand, Kijas *et al*.^33^ calculated genetic diversity of sheep by using two types of SNP chip data which were genotyped by Roche 454 and Illumina GA sequencing, respectively. The Roche 454 SNPs were developed primarily using animals of European origin, while the Illumina GA SNPs were designed using a larger number of animals selected from multiple regions. The results showed that ascertainment bias was unlikely to heavily influence genetic diversity between breeds and regions. From all the evidence presented above, we believe that ascertainment bias should have little influence on our results.

In conclusion, we show that native sheep and cattle breeds from developed regions in this study make a greater contribution to total genomic diversity than those from developing and underdeveloped regions; thus, a higher conservation priority should be given to these breeds. In contrast, most of the breeds in underdeveloped and developing regions make a small or no contribution at all to total genomic diversity and therefore receive relatively lower rankings in terms of conservation priority. Nevertheless, we noticed that breeds from underdeveloped countries tend to contribute more to between-breed diversity (Fig. 3). This is also a conservation objective, as many of these small, isolated and peripheral populations may have valuable variation (e.g., private alleles) that should be conserved. Also, it should be noted that the lack of samples in some distribution areas of sheep and cattle limit the generality of the results. The mechanisms underlying these observations are not well understood, but the regional annual GDP per capita and its growth rate, which are two important indicators of economic development, could be the missing link and help to explain the differences in the genomic diversity contributions of livestock breeds from different regions. In addition to genomic diversity, other factors, such as sociocultural value, local adaptation and the special uses of some livestock breeds, should also be considered in the development of conservation programs. With an awareness that the conservation recommendations made from the analysis of the contribution of breeds to diversity depend on the conservation interests and scopes, our results could help local authorities and animal breeders to ensure the effective and sustainable utilization of animal genetic resources in the future.

## Methods

The methods were carried out in accordance with the approved guidelines of the Good Experimental Practices adopted by the Institute of Zoology, Chinese Academy of Sciences. All experimental procedures and animal collections were conducted under a permit (No. IOZ13015) approved by the Committee for Animal Experiments of the Institute of Zoology, Chinese Academy of Sciences, China.

### Ovine sample collection and DNA extraction

Blood or tissue samples from 783 animals representing 40 native Chinese sheep breeds were collected in this study (Supplementary Table 1). In all cases, particular efforts were made, based on both pedigree information and the knowledge of local herdsmen, to ensure that the animals were unrelated and typical of their breeds. Whole blood samples were collected into evacuated tubes containing EDTA and ear marginal tissues were collected and stored in 2-ml microcentrifuge tubes containing 75% ethanol. Genomic DNA was extracted from the tissue samples using the standard phenol/chloroform protocol^49^ or from blood using the General AllGen Kit (Tiangen Biotech, Beijing, China) following the manufacturer’s instructions.

### Ovine SNP datasets genotyping

Of the 40 Chinese native sheep breeds/populations, 35 breeds (see Supplementary Table 1; Dataset I) were genotyped with the Illumina Ovine SNP50 (54,241 SNPs) BeadChip, and 5 breeds (Hu, Wadi, Sishui Fur, Large-tailed Han and Small-tailed Han sheep; see Supplementary Table 1; Dataset II) were genotyped with the Illumina Ovine Infinium HD (685,734 SNPs) BeadChip. The Ovine SNP50 and Infinium HD BeadChips were developed by the International Sheep Genomics Consortium (ISGC; http://www.sheephapmap.org). Details regarding SNP discovery, the design of the ovine array and genotyping procedures for the BeadChips can be found at the following address: http://www.sheephapmap.org/hapmap.php, and in Kijas *et al*.^33^ for the SNP50 BeadChip and Anderson *et al*.^50^ for the HD SNP BeadChip.

### Published Ovine SNP dataset

An Ovine SNP50 BeadChip dataset of 74 worldwide-distributed breeds/populations (Dataset III) was retrieved from a previous study^33^. A set of 57 native breeds (Supplementary Table 1) that had been sampled and genotyped within the Sheep HapMap project were selected out of the 74 breeds (for information on the breeds and their geographic origins, see Kijas *et al*.^33^).

### Ovine SNP datasets quality control

All of the ovine SNP BeadChip datasets (Datasets I, II and III) were merged to apply quality control procedures using Plink v.1.9 software^51^. The SNP quality control measures have been detailed elsewhere, in Kijas *et al*.^52^, Miller *et al*.^53^, Kijas *et al*.^33^ and Lv *et al*.^54^. Briefly, quality control was performed according the following criteria: (1) individuals with a call rate >95%, (2) SNPs with a minor allele frequency (MAF) >0.05, (3) SNPs with a >95% genotyping rate, and (4) SNPs with physical locations on autosomes. After removing the SNPs and individuals that failed to meet any of the above criteria, 39,641 SNPs and 1,910 individuals were retained in our working dataset.

### Bovine SNP datasets

Bovine SNP50 BeadChip datasets were retrieved from previous publications^10,34^, which included 53 native cattle breeds across its main distributions in the world (Supplementary Table 2). SNPs and individuals that failed to meet any of the following criteria were removed: (1) individuals with a call rate >95%, (2) MAF > 0.05, (3) SNPs with a >95% genotyping rate, and (4) SNPs with physical locations on autosomes (see also Decker *et al*.^34^). After filtering, there were 37,827 SNPs and 1,031 animals available for further analyses.

### Genetic diversity

The observed (*H*_O_) and expected (*H*_E_) heterozygosity of each sheep and cattle breed were estimated using Plink v.1.9^51^. They are important within-breed genetic diversity parameters for the conservation priorities in domesticated animals. In addition, *F*_IS_ was calculated by genepop R package^55^ based on the observed versus expected number of homozygous genotypes.

### Effective population size

Effective population size (*N*_e_) is a well-known parameter in evaluating conservation priorities for a species. Estimates of contemporary *N*_e_ were performed with the linkage disequilibrium method using NE ESTIMATOR v.2.1 software^56^ with the following parameter settings: random mating model and a Pcrit value with 0.05 which as the criterion for excluding rare alleles. In order to insure all loci in the analysis are physically unlinked^57^, we removed pairwise comparisons with *r*^2^ ≥ 0.05 before calculating *N*_e_ using Plink v.1.9 software^51^ (indep-pairwise 100 25 0.05). In addition, we divided the *N*_e_ value into three categories: *N*_e_ ≤ 100, 100 < *N*_e_ < 200, and *N*_e_ ≥ 200.

### Runs of homozygosity (ROH)

Runs of homozygosity (ROHs) were detected using the Plink v.1.9 software^51^ for each individual. The following settings were generally suitable for ROH identification in the domesticated animals^58^: (1) required minimum density (-homozyg-density 1000) and (2) number of heterozygotes allowed in a window (-homozyg-window-het 1). In this study, we detected ROH with the minimum length of 500 kb (-homozyg-window-kb 500) and a minimum SNP number of 50 SNPs (-homozyg-window-snp 50). The number (N_ROH_) and average length (L_ROH_) of ROH for each breed were estimated and the N_ROH_ and L_ROH_ were divided into three categories: N_ROH_ ≤ 100, 100 < N_ROH_ < 200, and N_ROH_ ≥ 200; L_ROH_ ≥ 20 Mb, 15 Mb < L_ROH_ < 20 Mb, and L_ROH_ ≤ 15 Mb. In addition, the inbreeding coefficient based on ROH (F_ROH_) was calculated for each animal according the following formula^26,59^:

1 $${F}_{ROH}=\frac{{\sum }_{K}(Length(RO{H}_{k}))}{L}$$

where the numerator represents the sum of ROH per animal above 500 kb length and L is the total length of genome. Total lengths of the cattle and sheep genomes were considered as 2,510,611 kb^60^ and 2,610,000 kb^61^, respectively. For sheep and cattle, the mean of F_ROH_ of each breed were estimated separately. The breed F_ROH_ values were divided into three ranges: F_ROH_ ≥ 0.10, 0.05 < F_ROH_ < 0.10, and F_ROH_ ≤ 0.05.

### Genetic structure analysis

In order to understand the relationship within and between breeds across each major geographic group, neighbor-joining (NJ) trees were constructed by the software SplitTree 4^62^. To avoid the SNPs with high levels of LD distorting the NJ tree, SNP were pruned using Plink v.1.9^51^. For the sheep and cattle dataset, the 39,641 and 37,827 SNPs were pruned to 34,770, and 29,093 SNPs respectively, by application of LD pruning command line–indep-pairwise 100 25 0.25. Subsequently, Reynolds genetic distances^63^ were calculated by Arlequin 3.5.2.2^64^, and the NJ trees were constructed using SplitsTree 4^62^ and visualized by FigTree v.1.3.1^65^.

### Analysis of conservation priorities based on the molecular coancestry approach

The method of molecular coancestry^8^ has proven to be efficient for defining conservation priorities for domestic animals. Unlike prioritization based on Weitzman diversity measures^66,67^, this method considers within-breed genetic variation, which is of great importance for the management of livestock^68^. The method assumes that there is a metapopulation including *n* populations with *N*_*i*_ breeding individuals. In the metapopulation, s_*i*_ denotes the average self-coancestry of the *N*_*i*_ individuals of population *i*; *D*_*ii*_ is the average distance between individuals of the *i*^th^ population; and *D*_*ij*_ is Nei’s minimum distance between populations *i* and *j*^69^. The average global coancestry ($\bar{f}$) was calculated as follows^70^:

2 $$\bar{f}=\sum _{i=1}^{n}\,\frac{1}{n}[{s}_{i}-{D}_{ii}-\frac{{\sum }_{j=1}^{n}\,{D}_{ij}}{n}]$$

This equation can also be expressed based on genetic diversity^70^:

3 $$(1-\bar{f})=(1-\tilde{f})+\bar{D}=\,1-\tilde{s}+\tilde{D}+\bar{D}=(1-\tilde{s})+(\tilde{s}-\tilde{f})+(\tilde{f}-\bar{f})$$

In equation (3), $\tilde{f}$ and $\tilde{s}$ are the mean coancestry and self-coancestry of the populations, respectively; $\tilde{D}$ is Nei’s minimum distance between individuals within populations; and $\bar{D}$ is the average genetic distance over the entire metapopulation. In the derived expression, the total genetic diversity (${{\rm{GD}}}_{{\rm{T}}}=1-\bar{f}$) was divided into three partitions: the genetic diversity within individuals (${{\rm{GD}}}_{{\rm{WI}}}=1-\tilde{s}$), the genetic diversity between individuals (${{\rm{GD}}}_{{\rm{BI}}}=\tilde{s}-\tilde{f}$) and the genetic diversity between populations (${{\rm{GD}}}_{{\rm{BS}}}=\tilde{f}-\bar{f}$). The sum of the first two components (GD_WI_ and GD_BI_) gives the genetic diversity within populations (${{\rm{GD}}}_{{\rm{WS}}}=1-\tilde{f}$). As a result, the total genetic diversity GD_T_ is assumed to be the sum of the genetic diversity within populations (GD_WS_) and the genetic diversity between populations (GD_BS_), i.e., GD_T_ = GD_WS_ + GD_BS_.

The loss or gain of genetic diversity (ΔGD_WS,_ ΔGD_BS_ and ΔGD_T_) was estimated using Metapop v.2.0 software^71^ when each of the populations was removed from the dataset. The parameter of *λ* = 1, which indicates equal weight for within- and between-breed diversity, was applied in the Metapop calculations^71^ (Supplementary Tables 1 and 2). It is noteworthy that the initial version of Metapop used loss (−) vs gain (+) of diversity as indicative of high vs low contribution of diversity, respectively. However, in the latest version of Metapop (used in this paper), the sign was changed to go to a more intuitive view: + means greater contribution and − means lower contribution.

### Analysis of conservation priorities based on the allelic richness approach

Allelic richness is an important measure of genetic diversity for setting conservation priorities in livestock. The rarefaction method developed by Hurlbert is advantageous for estimating allelic richness^72,73^. With this method, El Mousadik and Petit denoted allelic richness as *a*_*i*_, representing the number of different alleles in a sample of genes taken at random. The calculation formula is as follows^74^:

4 $${a}_{i}=\sum _{k=1}^{K}\,(1-{P}_{ik})=\sum _{k=1}^{K}(1-\frac{(\begin{array}{c}{N}_{i}-{N}_{ik}\\ {\rm{g}}\end{array})}{(\begin{array}{c}{N}_{i}\\ {\rm{g}}\end{array})})$$

where *P*_*ik*_ is the probability that allele k does not exist in a random sample of g genes; *N*_*i*_ represents the total number of genes in the sample of a given subpopulation i and *N*_*ik*_ represents the number of copies of the kth allele from that subpopulation.

The within-subpopulation component of allelic diversity was calculated according to the following formula^75^:

5 $${A}_{s}=(\frac{1}{n}\sum _{i=1}^{n}\,{a}_{i})-1$$

The average distance between all subpopulations is

6 $${D}_{A}=\frac{1}{{n}^{2}}[\sum _{i,j=1}^{n}\,{d}_{A,ij}]$$

where *d*_*A*,*ij*_ is the average allelic distance between subpopulations i and j, and it is equal to

7 $${d}_{A,ij}=\frac{1}{2}\sum _{k=1}^{K}[(1-{P}_{ik}){P}_{jk}+{P}_{ik}(1-{P}_{jk})]$$

The total allelic diversity is estimated with the following equation:

8 $${A}_{T}={A}_{S}+{D}_{A}=[\frac{1}{n}\sum _{i=1}^{n}({a}_{i}+\frac{1}{n}\sum _{i=1}^{n}\,{d}_{ij})]-1=[\frac{1}{{n}^{2}}\sum _{k=1}^{K}\,\sum _{i,j=1}^{n}\,(1-{P}_{ik}{P}_{jk})]-1$$

The three estimators (*A*_S_, *D*_A_ and *A*_T_) were calculated using Metapop v.2.0 software as described above (Supplementary Tables 1 and 2).

The results obtained by the molecular coancestry and allelic richness methods have some differences. However, we compared the estimated values based on both methods using paired *t*-tests, and found that they did not show significant differences for the three estimators (for sheep and cattle, ΔGD_WS_; *t* = 0.01, df = 96, *P* = 0.999 and *t* = 0.031, df = 52, *P* = 0.976; ΔGD_BS_: *t* = 0.2, df = 96, *P* = 0.842 and *t* = 0.286 df = 52, *P* = 0.776; and ΔGD_T_: *t* = 0.301 df = 96, *P* = 0.764 and *t* = 0.429, df = 52, *P* = 0.670, respectively). In order to obtain more accurate results, the average values of the estimates obtained using the two approaches were employed in the following statistical analysis. The average values of ΔGD_WS,_ ΔGD_BS_ and ΔGD_T_ in sheep and cattle were divided into four fractions: <−0.1, −0.1–0, 0–0.1 and >0.1, and then plotted around the world using the software ArcGIS v.10.1 (ESRI Inc, Redlands, CA, USA) (Supplementary Fig. 4).

### Analysis of the effect of regional economics on the conservation priority of different breeds

We further investigated the associations between the changes (loss or gain) in genetic diversity for each breed (or group of breeds) and the economies of the countries (or regions) from which the breeds originated. The average GDP per capita of the countries (or regions) during the years 1993 to 2013 was collected from the World Bank database (http://data.worldbank.org.cn/indicator). Because the 40 native Chinese sheep populations originated from 13 provinces, which differ greatly in their economies and employ different breeding and conservation strategies for domestic animals^76^, the GDP per capita for the provinces, rather than the whole country of China, was collected for the period from 1993 to 2013 (from the China Statistics Bureau; available at http://data.stats.gov.cn; Supplementary Table 5) and included in the analysis. The countries (or regions) were classified into three different categories based on their average GDP per capita (A-GDPPC) during these years: underdeveloped countries/regions (A-GDPPC ≤ $1,500); developing countries/regions ($1,500 ≤ A-GDPPC ≤ $15,000); and developed countries/regions (A-GDPPC ≥ $15,000). For sheep, the breeds came from 38 countries or regions: 9 were underdeveloped; 16 were developing; and 13 were developed. For cattle, the breeds came from 21 countries or regions: 10 were underdeveloped; 3 were developing; and 8 were developed (see Supplementary Tables 3 and 4). Subsequently, we calculated the loss or gain of diversity for each country/region when all of the breeds from the country/region were removed using the two approaches described above (Supplementary Tables 3 and 4). Differences in the loss or gain of genetic diversity averaged over sheep and cattle breeds in the three different economic categories were compared using the boxplots statistics in the boxplot package of *R* software v.3.4.3^77^, and the *P* value was calculated using the Kolmogorov-Smirnov test^78^.

To evaluate the effect of economic development on the loss or gain of genetic diversity for the breeds, we performed regression analysis between the loss or gain of genetic diversity (within subpopulation, ΔGD_WS_; between subpopulations, ΔGD_BS_; and total genetic diversity, ΔGD_T_) and the increased average GDP per capita (ΔGDPPC) for 1993 to 2013 from each country (or region) using the dual-regression approach. The coefficient of determination (*R*^2^) and its significance (*p*-value) were calculated using SPSS v.22.0 software^79^.

### Data availability

The data sets analyzed in the present study are available from the corresponding author on reasonable request.

## Electronic supplementary material

Supplementary Information

Supplementary Table 1

Supplementary Table 2

Supplementary Table 3

Supplementary Table 4

Supplementary Table 5

## References

[1] Hodges J (1999). Jubilee history of the European Association for animal production: 1949–1999. Livest. Prod. Sci..

[2] FAO. The second report on the state of the world’s animal genetic resources for food and agriculture. In *FAO Commission on Genetic Resources for Food andAgriculture Assessments* (eds. Scherf, B. D. & Pilling, D) (Food & Agriculture Organization, Rome, 2015).

[3] Hoffmann I, Boerma D, Scherf B (2011). The Global Plan of Action for Animal Genetic Resources—The road to common understanding and agreement. Livest. Sci..

[4] Rook A (2004). Matching type of livestock to desired biodiversity outcomes in pastures–a review. Biol. Conserv..

[5] Lupton CJ (2008). Impacts of animal science research on United States sheep production and predictions for the future. J. Anim. Sci..

[6] Tisdell C (2003). Socioeconomic causes of loss of animal genetic diversity: analysis and assessment. Ecol. Econ..

[7] Drucker AG, Anderson S (2004). Economic analysis of animal genetic resources and the use of rural appraisal methods: lessons from Southeast Mexico. Int. J. Agr. Sustain..

[8] Caballero A, Toro MA (2002). Analysis of genetic diversity for the management of conserved subdivided populations. Conserv. Genet..

[9] Petit RJ, El Mousadik A, Pons O (1998). Identifying populations for conservation on the basis of genetic markers. Converv. Biol..

[10] Groeneveld L (2010). Genetic diversity in farm animals–a review. Anim. Genet..

[11] The Bovine HapMap Consortium (2009). Genome-wide survey of SNP variation uncovers the genetic structure of cattle breeds. Science.

[12] Chessa B (2009). Revealing the history of sheep domestication using retrovirus integrations. Science.

[13] Lv F-H (2015). Mitogenomic meta-analysis identifies two phases of migration in the history of Eastern Eurasian sheep. Mol. Biol. Evol..

[14] Loftus RT, MacHugh DE, Bradley DG, Sharp PM, Cunningham P (1994). Evidence for two independent domestications of cattle. Proc. Natl. Acad. Sci. USA.

[15] McTavish EJ, Decker JE, Schnabel RD, Taylor JF, Hillis DM (2013). New World cattle show ancestry from multiple independent domestication events. Proc. Natl. Acad. Sci. USA.

[16] Franklin, I. R. Evolutionary change in small populations. In *Conservation Biology: An Evolutionary—Ecological Perspective* (eds Soulé, M. E. & Wilcox, B. A.), 135–149 (Sinauer Associates, Sunderland, MA, 1980).

[17] Frankham R, Bradshaw CJ, Brook BW (2014). Genetics in conservation management: revised recommendations for the 50/500 rules, Red List criteria and population viability analyses. Biol. Conserv..

[18] RBST. http://www.rbst.org.uk/Our-Work/watchlist-overview (2018).

[19] Wiltshire Horn. https://en.wikipedia.org/wiki/Wiltshire_Horn (2018).

[20] Coulson T (2001). Age, sex, density, winter weather, and population crashes in Soay sheep. Science.

[21] RBST. http://www.rbst.org.uk/soay (2018).

[22] Talib C, Entwistle K, Siregar A, Budiarti-Turner S, Lindsay D (2003). Survey of population and production dynamics of Bali cattle and existing breeding programs in Indonesia. ACIAR Proc..

[23] Gibson J, Morton NE, Collins A (2006). Extended tracts of homozygosity in outbred human populations. Hum. Mol. Genet..

[24] Keller MC, Visscher PM, Goddard ME (2011). Quantification of inbreeding due to distant ancestors and its detection using dense single nucleotide polymorphism data. Genetics.

[25] Bosse M (2012). Regions of homozygosity in the porcine genome: consequence of demography and the recombination landscape. Plos Genet..

[26] McQuillan R (2008). Runs of homozygosity in European populations. Am. J. Hum. Genet..

[27] Ouborg NJ, Pertoldi C, Loeschcke V, Bijlsma RK, Hedrick PW (2010). Conservation genetics in transition to conservation genomics. Trends Genet..

[28] Leroy G (2014). Inbreeding depression in livestock species: review and meta-analysis. Anim. Genet..

[29] Garcia-Gamez E, Sahana G, Gutierrez-Gil B, Arranz JJ (2012). Linkage disequilibrium and inbreeding estimation in Spanish Churra sheep. BMC Genet..

[30] Kim ES (2013). Effect of artificial selection on runs of homozygosity in U.S. Holstein cattle. Plos One.

[31] Kim ES, Sonstegard TS, Van Tassell CP, Wiggans G, Rothschild MF (2015). The relationship between runs of homozygosity and inbreeding in Jersey cattle under selection. Plos One.

[32] Ferencakovic M (2013). Estimates of autozygosity derived from runs of homozygosity: empirical evidence from selected cattle populations. J. Anim. Breed. Genet..

[33] Kijas JW (2012). Genome-wide analysis of the world’s sheep breeds reveals high levels of historic mixture and strong recent selection. Plos Biol..

[34] Decker JE (2014). Worldwide patterns of ancestry, divergence, and admixture in domesticated cattle. Plos Genet..

[35] Hiemstra, S. J., Haas, Y., Mäki-Tanila, A. & Gandini, G. Local cattle breeds in Europe: Development of policies and strategies for self-sustaining breeds (Wageningen Academic Publishers, 2010).

[36] Baylis K, Peplow S, Rausser G, Simon L (2008). Agri-environmental policies in the EU and United States: A comparison. Ecol. Econ..

[37] Gaspar P, Mesías F, Escribano M, Pulido F (2009). Assessing the technical efficiency of extensive livestock farming systems in Extremadura, Spain. Livest. Sci..

[38] Taberlet P (2008). Are cattle, sheep, and goats endangered species?. Mol. Ecol..

[39] Gandini G (2010). Motives and values in farming local cattle breeds inEurope: a survey on 15 breeds. Anim. Genet. Resour..

[40] Blackburn H (2009). Genebank development for the conservation of livestock genetic resources in the United States of America. Livest. Sci..

[41] Samson F, Knopf F (1994). Prairie conservation in north america. BioScience.

[42] Åby B, Aass L, Sehested E, Vangen O (2012). Effects of changes in external production conditions on economic values of traits in Continental and British beef cattle breeds. Livest. Sci..

[43] Hiemstra S (2007). What’s on the menu? Options for strengthening the policy and regulatory framework for the exchange, use and conservation of animal genetic resources. Anim. Genet. Resour..

[44] Gollin D, Van Dusen E, Blackburn H (2009). Animal genetic resource trade flows: Economic assessment. Livest. Sci..

[45] Hoffmann I (2010). Livestock biodiversity. Rev. Sci. Tech..

[46] Hoffmann I (2010). Climate change and the characterization, breeding and conservation of animal genetic resources. Anim. Genet..

[47] Huo J (2014). Genetic diversity of local Yunnan chicken breeds and their relationships with Red Junglefowl. Genet. Mol. Res..

[48] López Herráez D (2009). Genetic variation and recent positive selection in worldwide human populations: evidence from nearly 1 million SNPs. Plos One.

[49] Sambrook, J. & Russel, D. W. *Molecular Cloning: A Laboratory Manual 3rd edn* (Cold Spring Harbor Laboratory Press, Cold Spring Harbor, 2001).

[50] Anderson, R. *et al*. Development of a High Density (600 K) Illumina Ovine SNP Chip and Its Use to Fine Map the Yellow Fat Locus. In *Plant and Animal Genome XXII Conference* (Plant and Animal Genome, San Diego, 2014).

[51] Purcell S (2007). PLINK: a tool set for whole-genome association and population-based linkage analyses. Am. J. Hum. Genet..

[52] Kijas JW (2009). A genome wide survey of SNP variation reveals the genetic structure of sheep breeds. Plos One.

[53] Miller JM, Poissant J, Kijas JW, Coltman DW (2011). & International Sheep Genomics Consortium. A genome-wide set of SNPs detects population substructure and long range linkage disequilibrium in wild sheep. Mol. Ecol. Resour..

[54] Lv F-H (2014). Adaptations to climate-mediated selective pressures in sheep. Mol. Biol. Evol..

[55] Rousset F (2008). Genepop’007: a complete re-implementation of the genepop software for Windows and Linux. Mol. Ecol. Resour..

[56] Do C (2014). NEESTIMATOR v2: re-implementation of software for the estimation of contemporary effective population size (N*e*) from genetic data. Mol. Ecol. Resour..

[57] Waples RK, Larson WA, Waples RS (2016). Estimating contemporary effective population size in non-model species using linkage disequilibrium across thousands of loci. Heredity.

[58] Zhao Y-X (2017). Genomic reconstruction of the history of native sheep reveals the peopling patterns of nomads and the expansion of early pastoralism in East Asia. Mol. Biol. Evol..

[59] Karimi K, Koshkoiyeh AE, Fozi MA, Porto-Neto LR, Gondro C (2016). Prioritization for conservation of Iranian native cattle breeds based on genome-wide SNP data. Conserv. Genet..

[60] Purfield DC, Berry DP, McParland S, Bradley DG (2012). Runs of homozygosity and population history in cattle. BMC Genet..

[61] Jiang Y (2014). The sheep genome illuminates biology of the rumen and lipid metabolism. Science.

[62] Perl Y (1984). Optimum split trees. J. Algorithms.

[63] Reynolds J, Weir BS, Cockerham CC (1983). Estimation of the coancestry coefficient: basis for a short-term genetic distance. Genetics.

[64] Excoffier L, Lischer HE (2010). Arlequin suite ver 3.5: a new series of programs to perform population genetics analyses under Linux and Windows. Mol. Ecol. Resour..

[65] Rambaut, A. & Drummond, A. FigTree v1.3. 1. *Institute of Evolutionary Biology*, *University of Edinburg*h (2010).

[66] Weitzman ML (1992). On diversity. Q. J. Econ..

[67] d’Arnoldi CT, Foulley J-L, Ollivier L (1998). An overview of the Weitzman approach to diversity. Genet. Sel. Evol..

[68] Ollivier L, Foulley J-L (2005). Aggregate diversity: new approach combining within-and between-breed genetic diversity. Livest. Prod. Sci..

[69] Cockerham CC (1967). Group inbreeding and coancestry. Genetics.

[70] Fabuel E, Barragán C, Silió L, Rodriguez M, Toro M (2004). Analysis of genetic diversity and conservation priorities in Iberian pigs based on microsatellite markers. Heredity.

[71] Pérez-Figueroa A, Saura M, Fernández J, Toro MA, Caballero A (2009). METAPOP—A software for the management and analysis of subdivided populations in conservation programs. Conser. Genet..

[72] Sanders HL (1968). Marine benthic diversity: a comparison study. Am. Nat..

[73] Hurlbert SH (1971). The nonconcept of species diversity: a critique and alternative parameters. Ecology.

[74] El Mousadik A, Petit RJ (1996). High level of genetic differentiation for allelic richness among populations of the argan tree [*Argania spinosa* (L.) Skeels] endemic to Morocco. Theor. Appl. Genet..

[75] Caballero A, Rodríguez-Ramilo ST (2010). A new method for the partition of allelic diversity within and between subpopulations. Conserv. Genet..

[76] Huang D, Wang K, Wu W (2007). Problems and strategies for sustainable development of farming and animal husbandry in the Agro-Pastoral Transition Zone in Northern China (APTZNC). Int. J. Sust. Dev. World Ecol..

[77] R Development Core Team. R: A language and environment for statistical computing. *R Foundation for Statistical Computing*, *Vienna* (2018).

[78] Gail MH, Green SB (1976). A generalization of the one-sided two-sample Kolmogorov-Smirnov statistic for evaluating diagnostic tests. Biometrics.

[79] Mather LE, Austin KL (1983). The Statistical Package for the Social Sciences (SPSS) as an adjunct to pharmacokinetic analysis. Biopharm. Drug Dispos..

