# Supplementary material for: Global genomic diversity and conservation priorities for domestic animals are associated with the economies of their regions of origin
Source: Sci Rep. 2018 Aug 3;8:11677. doi: 10.1038/s41598-018-30061-0 (PMC6076285; doi:10.1038/s41598-018-30061-0)
Supplement: Supplementary file 1 — Supplementary Information [file 41598_2018_30061_MOESM1_ESM.pdf]

Global genomic diversity and conservation priorities for domestic animals are associated with the economies of their regions of origin

Min Zhang[1,2], Wei-Feng Peng[2,3], Xiao-Ju Hu[2,3], Yong-Xin Zhao[2,3], Feng-Hua Lv[2] & Ji Yang[2]

[1]School of Life Sciences, University of Science and Technology of China, Hefei, Anhui 230026, China

[2]CAS Key Laboratory of Animal Ecology and Conservation Biology, Institute of Zoology, Chinese Academy of Sciences (CAS), Beijing 100101, China

[3]University of Chinese Academy of Sciences (UCAS), Beijing 100049, China

Correspondence and requests for materials should be addressed to J.Y. (yangji@ioz.ac.cn)

Supplementary Figures

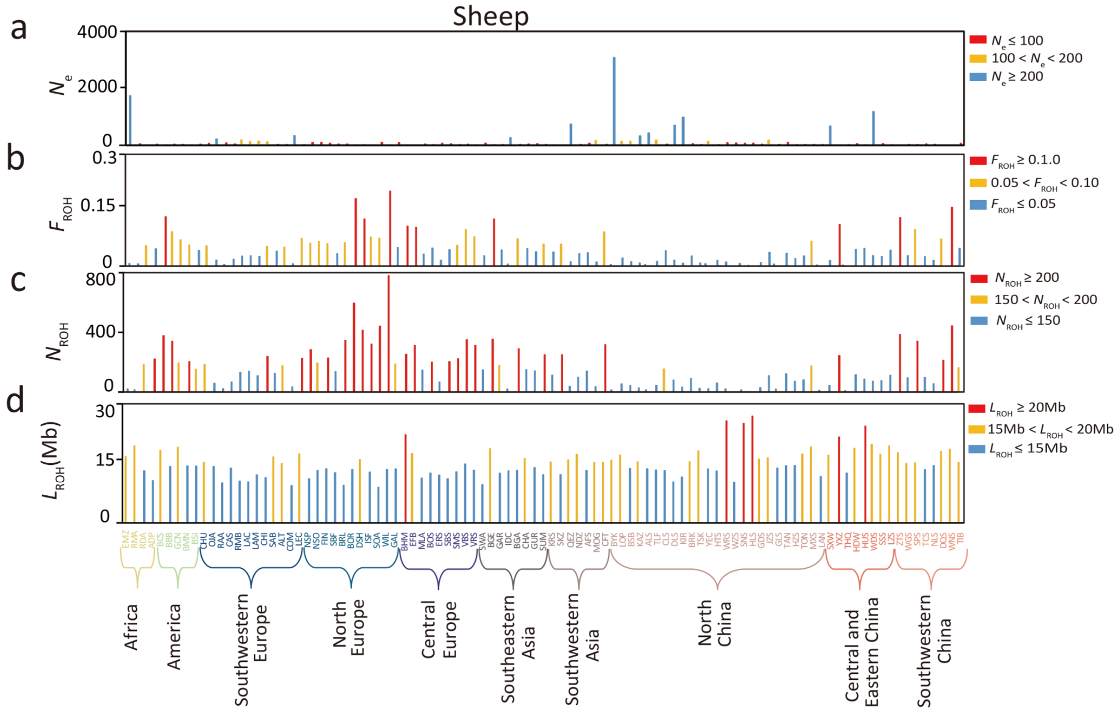

Supplementary Figure 1. Distribution patterns of N_e (a), NROH (b), LROH (c), FROH (d) found in 97 sheep breeds. N_e: effective population size. FROH: inbreeding coefficient based on runs of homozygosity. NROH: number of ROH. LROH: mean length of ROH.

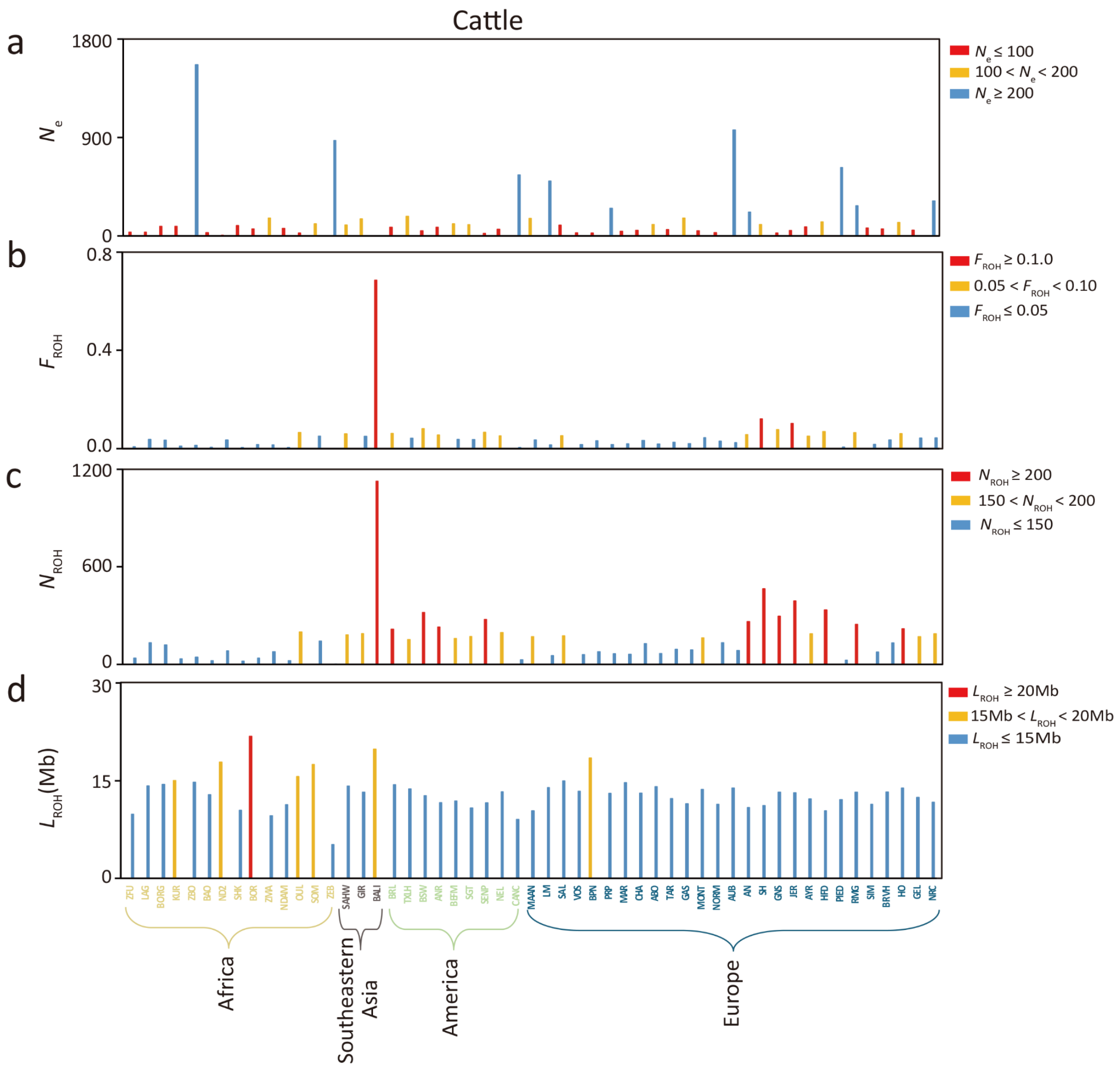

Supplementary Figure 2. Distribution patterns of N_e (a), N_{ROH} (b), L_{ROH} (c), F_{ROH} (d) found in 53 cattle breeds. N_e: effective population size. F_{ROH}: inbreeding coefficient based on runs of homozygosity. N_{ROH}: number of ROH. L_{ROH}: mean length of ROH.

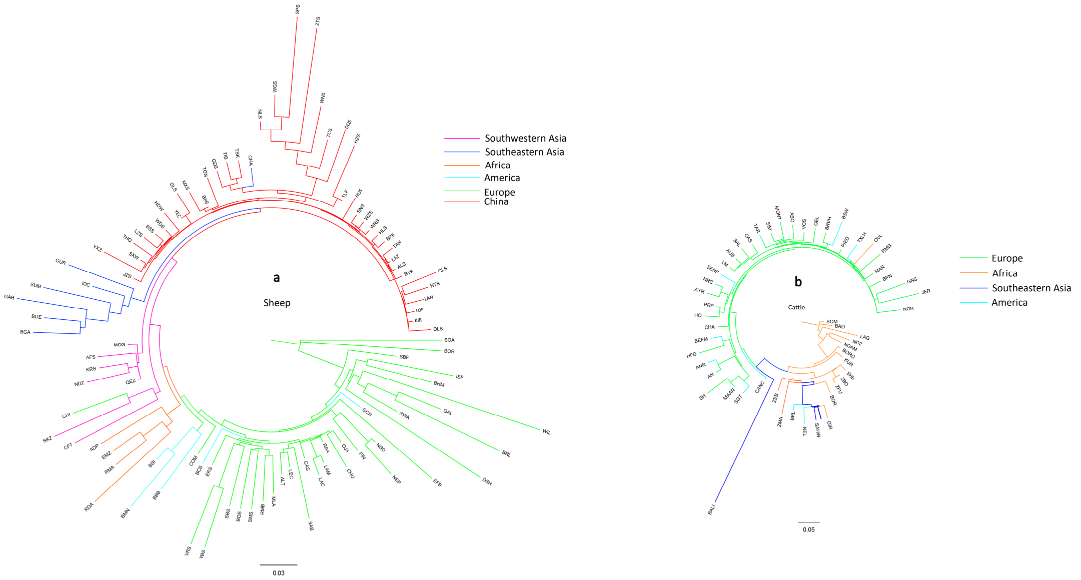

Supplementary Figure 3. Relationship of sheep (a) and cattle (b) breeds based on Reynolds distance. An allele frequency-dependent distance metric (Reynolds) was used to construct a Neighbor Net graph relating breeds.

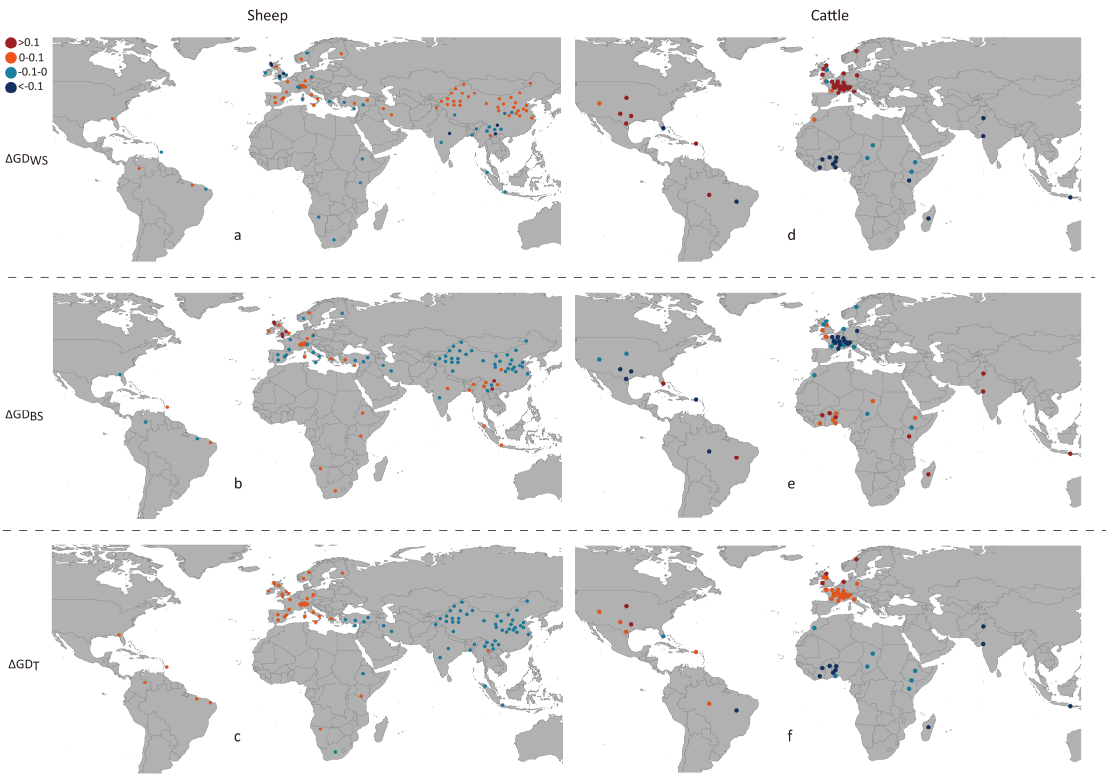

Supplementary Figure 4. Loss or gain of genetic diversity in each breed of sheep and cattle. (a to c) The gain or loss of genomic diversity (ΔGD_{WS}, ΔGD_{WS}, and ΔGD_{T}) in sheep; (d to f) The gain or loss of genomic diversity (ΔGD_{WS}, ΔGD_{WS}, and ΔGD_{T}) in cattle. All of these values are divided into four ranges: < -0.1, -0.1-0, 0-0.1 and > 0.1. The maps were generated by the software ArcGIS 10.1 (ESRI Inc, Redlands, CA, USA, http://resources.arcgis.com/en/home/).

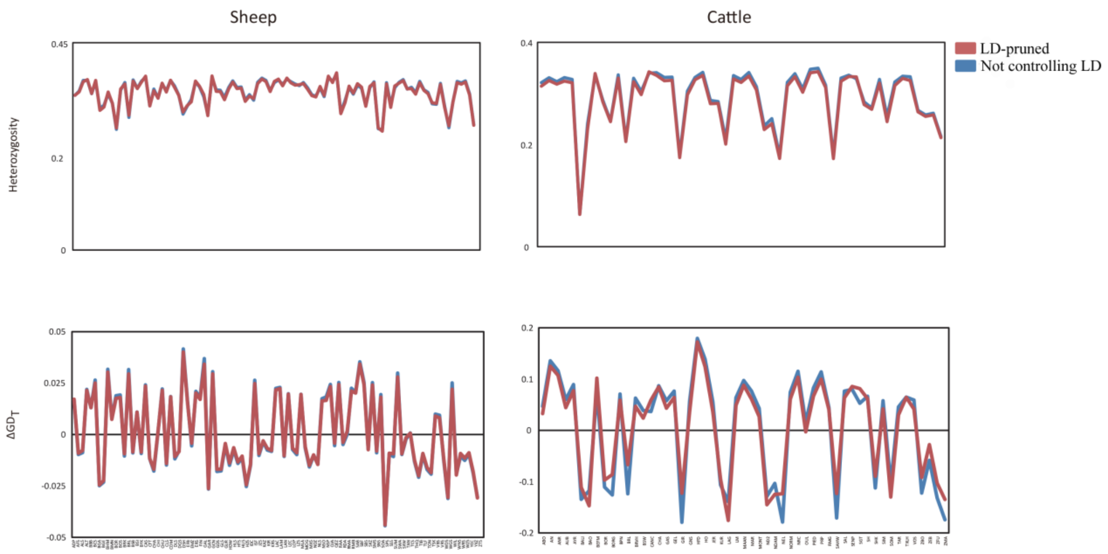

Supplementary Figure 5. The comparison of heterozygosity and *ΔGD*$_T$ in each breed of sheep and cattle between the LD-pruned data and the data not controlling for LD.

Supplementary Tables

Supplementary Table 1. Summary information of genetic diversity parameters and their loss or gain of genetic diversity (ΔGD_{WS} = loss or gain of gene diversity within subpopulations, ΔGD_{BS} = loss or gain of gene diversity between subpopulations, and ΔGD_{T} = loss or gain of total diversity) when the breed is removed in sheep. (Uploaded as excel file)

Supplementary Table 2. Summary information of genetic diversity parameters and their loss or gain of genetic diversity (ΔGD_{WS} = loss or gain of gene diversity within subpopulations, ΔGD_{BS} = loss or gain of gene diversity between subpopulations, and ΔGD_{T} = loss or gain of total diversity) when the breed is removed in cattle. (Uploaded as excel file)

Supplementary Table 3. Loss or gain of genetic diversity of sheep breeds (ΔGD_{WS} = loss or gain of gene diversity within subpopulations; ΔGD_{BS} = loss or gain of gene diversity between subpopulations; ΔGD_{T} = loss or gain of total diversity) when the breeds in the countries or regions are removed. (Uploaded as excel file)

Supplementary Table 4. Loss or gain of genetic diversity of cattle breeds (ΔGD_{WS} = loss or gain of gene diversity within subpopulations; ΔGD_{BS} = loss or gain of gene diversity between subpopulations; ΔGD_{T} = loss or gain of total diversity) when the breeds in the countries or regions are removed. (Uploaded as excel file)

Supplementary Table 5. GDP per capita of the countries or regions from 1993 to 2013 ($/capita). (Uploaded as excel file)
